# Young Adults, Social Networks, and Addiction Recovery: Post Treatment Changes in Social Ties and Their Role as a Mediator of 12-Step Participation

**DOI:** 10.1371/journal.pone.0100121

**Published:** 2014-06-19

**Authors:** John F. Kelly, Robert L. Stout, M. Claire Greene, Valerie Slaymaker

**Affiliations:** 1 Center for Addiction Medicine Departments of Psychiatry Massachusetts General Hospital and Harvard Medical School, Boston, Massachusetts, United States of America; 2 Pacific Institute for Research and Evaluation, Providence, Rhode Island, United States of America; 3 Hazelden Foundation, Center City, Minnesota, United States of America; California Pacific Medicial Center Research Institute, United States of America

## Abstract

**Background:**

Social factors play a key role in addiction recovery. Research with adults indicates individuals with substance use disorder (SUD) benefit from mutual-help organizations (MHOs), such as Alcoholics Anonymous, via their ability to facilitate adaptive network changes. Given the lower prevalence of sobriety-conducive, and sobriety-supportive, social contexts in the general population during the life-stage of young adulthood, however, 12-step MHOs may play an even more crucial recovery-supportive social role for young adults, but have not been investigated. Greater knowledge could enhance understanding of recovery-related change and inform young adults’ continuing care recommendations.

**Methods:**

Emerging adults (N = 302; 18–24 yrs; 26% female; 95% White) enrolled in a study of residential treatment effectiveness were assessed at intake, 1, 3, 6, and 12 months on 12-step attendance, peer network variables (“high [relapse] risk” and “low [relapse] risk” friends), and treatment outcomes (Percent Days Abstinent; Percent Days Heavy Drinking). Hierarchical linear models tested for change in social risk over time and lagged mediational analyses tested whether 12-step attendance conferred recovery benefits via change in social risk.

**Results:**

High-risk friends were common at treatment entry, but decreased during follow-up; low-risk friends increased. Contrary to predictions, while substantial recovery-supportive friend network changes were observed, this was unrelated to 12-step participation and, thus, not found to mediate its positive influence on outcome.

**Conclusions:**

Young adult 12-step participation confers recovery benefit; yet, while *encouraging* social network change, 12-step MHOs may be less able to *provide* social network change directly for young adults, perhaps because similar-aged peers are less common in MHOs. Findings highlight the importance of both social networks and 12-step MHOs and raise further questions as to how young adults benefit from 12-step MHOs.

## Introduction

From myriad theoretical standpoints social variables play a key role in the etiology and resolution of substance-related problems, and in relapse to substance use disorder (SUD) [1]–[4]. Research too shows social forces can wield a commanding influence on a variety of behavioral health trajectories and outcomes, including alcohol and other drug use [5]–[9]. SUD recovery often requires monitoring and management over the long-term [10], [11] and individuals suffering from SUD exist in a complex network of social forces that, in contrast to the short-term effects of formal care, exert a more enduring influence [12].

Successful recovery from SUD often involves changing social networks from those that are supportive of substance use to ones that are supportive of abstinence and recovery [13]–[16]. This change can reduce exposure to substance-related cues and facilitate the acquisition of recovery coping skills and abstinence self-efficacy that mitigate stress-related relapse risk [15], [17]. Given the strong association between social factors and SUD recovery, treatment providers encourage patients to make adaptive changes in their social networks and frequently refer patients to mutual-help organizations (MHOs), such as Alcoholics Anonymous (AA) and Narcotics Anonymous (NA), to help facilitate such change [18]–[20]. Research among adult SUD samples supports this clinical recommendation showing that participation in AA/NA leads to better substance use outcomes, in large part, by facilitating recovery supportive social changes in the networks of attendees [21]–[25]. Little is known, however, regarding young adults who face different social risks and recovery challenges associated with their life-stage context [19], [26].

A life-course perspective is important to consider as different life stages confer differing levels of protection or risk for a variety of disorders [27]. These developmental factors are relevant to SUD recovery since sobriety conducive contexts naturally become more prevalent in the general population as individuals age and transition into middle adulthood where rates of illicit drug use as well as alcohol/heavy alcohol use decrease [28]. In contrast, in most industrialized nations, young adulthood represents the life stage wherein the *highest* rates of alcohol and other drug use occur. Young adults suffering from SUD who are seeking recovery, therefore, may face additional recovery barriers since sobriety conducive and supportive peers and contexts may be at more of a premium. MHOs, such as AA and NA, consequently may be of greater value as a locatable venue for meeting recovering same-aged peers with whom new friendship can be made that ease and support the transition into a recovery lifestyle [25], [29], [30].

While prior research supports young adult participation in 12-step MHOs [19], [25], [30]–[32], compared to older adults, little is known regarding whether AA and NA facilitate youth recovery via this social mechanism. This is important to determine as mechanisms by which MHOs aid recovery may differ for young people. Blonigan and colleagues (2012) in a broad mixed-age sample of SUD patients found that impulsivity was reduced as a function of 12-step participation, but only among young adults [33]. Also, some of the intrapersonal mechanisms through which 12-step MHOs have been shown to aid adult recovery is through enhancing coping, self-efficacy, and abstinence motivation [34]. In adolescent samples, however, during the same early phase of recovery post-treatment, we have found that AA/NA aids recovery more by enhancing and maintaining motivation for abstinence and not coping and self-efficacy [35], [36]. The principal aim of this study, therefore, was to test whether one of the key mechanisms through which AA and NA has been shown to work in adult samples (i.e., via social network changes) is similar for young adults. Specifically, we examine whether AA/NA leads to better post-treatment outcomes by reducing high risk drinkers/drug users and increasing low risk users/abstainers, as has been shown in adults [22], [37].

## Methods

### Ethics Statement

This study was approved by and conducted in accordance with the Institutional Review Board at Schulmann Associates IRB, an independent review board, and all participants signed informed consent documents.

### Participants

Participants were 302 young adults (18–24 years old) undergoing residential treatment and enrolled in an observational study of treatment process and outcome. At intake, participants were 20.4 years old on average (*SD* = 1.6). Most were Caucasian (95.0%), male (73.8%), and all were single; 24.2% were employed full- or part-time, 31.8% were students; 43% of the sample obtained a high school diploma and 39.7% had some college education. The most commonly reported primary substance was alcohol (28.1%) and marijuana (28.1%), followed by heroin or other opiates (22.2%), cocaine or crack (12.3%), and amphetamines (6.0%). Small proportions reported benzodiazepines (2.0%), hallucinogens (1.0%), or ecstasy (1.0%) as their primary substance. A small number of participants (n = 5) reported more than one primary substance, such that these proportions do not sum to 100%.

### Treatment

Treatment was based on a 12-step philosophy of recovery, but also included motivational enhancement, cognitive-behavioral, and family therapy. Programming included clinical assessment, individual and group therapy, and a host of specialty groups tailored to meet the needs of individual clients (e.g relapse prevention, anger management). Integrated mental health care was available, including therapy and medication management. Participants’ average length of stay was 25.5 days (SD = 5.7). The majority (83.8%) were completed treatment.

### Procedure

Participants were enrolled in the study shortly after admission. A total of 607 young adults were admitted to treatment during the recruitment period (October 2006 to March 2008). All of those aged 21–24 years old were approached for study enrollment, as well as every second individual aged 18–20. This was done to ensure sufficient representation of the older age group, given the predominance of those aged 18–20 at the facility. Of those approached (*n = *384), 64 declined or withdrew participation. Following enrollment, an additional 17 participants withdrew prior to baseline assessment and the consent for one participant was misplaced. The final sample of 302 represents 78.6% of those approached for participation (see Kelly, Stout and Slaymaker, 2012 for more details; [38]).

Research staff conducted assessments at baseline, 1, 3, 6, and 12 months post-discharge. Each assessment included an interview portion, completed either in person or by telephone, and self-administered surveys. Participants were reimbursed $30 for the baseline assessment, and $20, $30, $40, and $50 for the post-treatment assessments at 1, 3, 6 and 12 months, respectively. Post-discharge, study retention rates were 84.5% (*n = *256) at 1-month follow-up, 81.8% (*n = *248) at 3-month follow-up, 74.3% (*n = *225) at 6-month follow-up, and 71.3% (*n = *216) at 12-month follow-up. Assessment completers were compared to non-completers on demographic, clinical, and substance use variables. Relative to those with post-secondary education, those with a high school education or less were more likely to be missed at all time points and was retained as a control variable.

### Measures

#### Form-90

The Form-90 [39], [40] is an interview-based measure capturing substance use information. The recall period for the baseline interviews was 90 days. Modifications were made to subsequent assessments to capture the entire time period elapsed since previous interview (e.g., 180 days at 12 m follow-up). Primary outcome measures derived from the Form-90 included percentage of days abstinent (PDA) from all substances (except nicotine), and percentage of days of heavy drinking (PDHD), defined as 6 or more drinks. The Form-90 has demonstrated good test-retest reliability and validity [41], [42].

#### Stages of Change Readiness and Treatment Eagerness Scale (SOCRATES)

The SOCRATES [43] is a self-report measure of motivation to change substance use, with items repeated separately for alcohol and other drugs and possesses 3 subscales: problem recognition, ambivalence, and taking steps. The items are rated on a 5-point Likert scale from *strongly disagree* (1) to *strongly agree* (5). Responses are summed to provide total scores for each subscale (potential ranges = 7–35, 4–20, and 8–40). The subscales have demonstrated acceptable to high internal consistency (α’s = .60–.85) and high test-retest reliability (ICC’s = .82–.94) among adults [43], with additional evidence of concurrent and predictive validity among adolescents [44].

#### Social Support Questionnaire (SSQ)

The SSQ [45], modified to include items assessing the alcohol and drug use patterns of key significant others [46], was used to assess the perceived availability of social support. The resulting measure, completed via interview, identified key social network members (i.e., close friends), as well as each member’s substance use status rated as one of the following: “*currently abstaining”*, *“infrequently uses”*, *“regularly uses”, “possibly abuses” “abuses”*). Participants were asked specifically to list up to five of their closest friends, as well as these friends’ alcohol/drug using status.

#### Commitment to Sobriety (CSS)

The CSS is a 5-item self-report measure assessing level of commitment to alcohol and drug abstinence (e.g., “Staying sober is the most important thing in my life”). Each is rated on a 6-point Likert scale from *strongly disagree* (1) to *strongly agree* (6). This measure shows good internal consistency (α’s = .89–.95), as well as criterion, convergent, and discriminant validity [47].

#### Mutual-Help Attendance

The Multidimensional Mutual-help Activity Scale is a comprehensive 32-item, interview-based index used to assess the array of potential 12-step activities including frequency of attendance at 12-step meetings. Interviews captured the entire time period elapsed since the previous interview. This measure has shown to have excellent psychometric properties showing high internal consistency and validity [36].

### Analysis Plan

#### Social Support Measurement

For purposes of data analysis, we classified peers as high risk vs. low risk based on their use of substances. Those who were reported by the patient as “regular users”, “possible abusers”, or “abusers”, of alcohol/drugs were classified as “high risk”, those who were reported by the patient as “infrequent users” or “abstainers” were classified as “low risk”. We also examined the patients’ rating of how supportive a given significant individual in their social network was to their recovery as a means to further classify network members as high or low risk; however, subjects reported the overwhelming majority of their network members to be supportive of recovery, so this measure was not pursued further.

#### Social Indicator Analyses

We used means and standard deviations to describe the frequency of low-risk and high-risk peers at all time points. We used hierarchical linear modeling (HLM) for repeated measures to analyze changes from baseline through month 12 in the number of high-risk and low-risk persons, with a separate analysis for high and low risk [48]. The only predictor in the model was a categorical time variable, and we tested for significant changes between all pairs of time points.

#### Outcome Measurement

We chose two primary outcome variables: (1) percent of days abstinent from alcohol and all illegal drugs [PDA], and (2) percent of days of heavy drinking [PDHD]. In preliminary analyses, we found these measures to be skewed, and therefore transformed these variables with a log transformation for PDA (log(1+PDA)), and a reciprocal transformation for PDHD (−1/(1+PDHD)).

#### Outcome and Mediation Analyses

**Figure 1:**
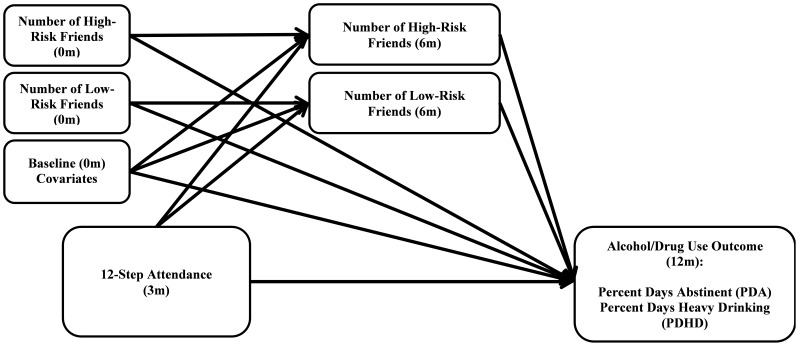
Lagged, Controlled, Mediational Model. Baseline covariates include gender, education, commitment to sobriety, prior SUD hospitalization, meeting with other 12-step group members outside of meetings, and baseline levels of alcohol/drug outcomes (PDA/PDHD).

The conceptual model guiding the outcome analyses is shown in Figure 1. In order to do fully prospective tests of mediation of the effects of 12-step attendance on mediators and outcome, we measured 12-step attendance at month 3, the proposed social support mediators at month 6, and substance use outcome was assessed at month 12. Because of missing data potentially related to outcomes, we chose to do multiple imputation [49] for missing values, using 50 replications. We did not impute missing substance use outcomes. Separate multiple linear regressions were computed for each leg of the A–B–C mediation paradigm: (1) the A–B leg in which 12-step attendance is used to predict number of high/low-risk friends, (2) the B–C leg where the number of friends is used to predict substance use outcome, and (3) the A–C leg, which is where we establish if 12-step attendance predicts outcomes without considering the mediator. We took precautions to ensure that the same subjects were represented in each of the three regressions. The mediation regressions were done a total of four times, allowing us to test for mediation of the effects of 12-step attendance by both high-risk and low-risk friend relationships, crossed with two dependent variables. All regressions controlled for predictors of attrition (education), baseline levels of the dependent variable, baseline levels of the mediator, and predictors of substance use outcome used in earlier analyses of this sample (i.e., age, gender, commitment to sobriety, motivation, prior SUD hospitalization and meeting with other 12-step members outside of meetings at baseline; [38]. Because the regressions that included the mediator variables (high/low-risk friends at month 6) covaried the baseline values of these variables, the residual change from baseline to month 6 is the mediating variable. The regression coefficients from the A–B and B–C regressions were then multiplied, and their significance tested using methods by MacKinnon [50].

## Results

### Social Support Changes Over Time

**Figure 2:**
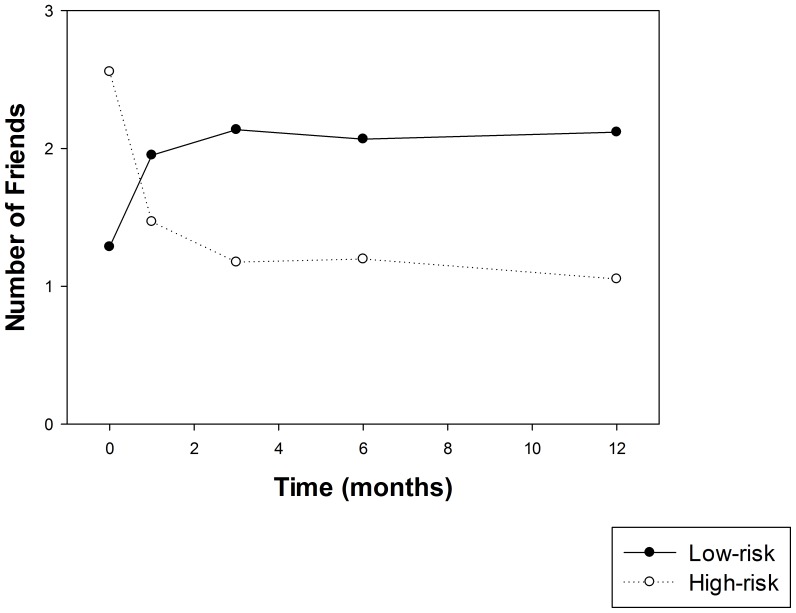
High vs. low-risk relationships over the 12 month study follow-up period. Note: Error bars indicate 95% confidence interval.

Descriptive statistics and tests for social support change over time are presented in Table 1 and Figure 2. The F test in Table 1 is for an overall effect of time on the number of high/low risk network members. Overall, where there is a significant effect of time, the number of high-risk friends declines substantially from baseline to all follow-up points, while the number of low-risk friends rises (results of comparisons of all pairs of time points are not shown for the sake of brevity) see Figure 2.

**Table 1:** Social network changes over follow-up period.

	High-Risk				Low-Risk			
	Mean number	SD	F_time_	*p*	Mean number	SD	F_time_	*p*
Friends			69.21	0.000			18.25	0.000
*Baseline*	2.556	0.089			1.285	0.092		
*1 Mo.*	1.469	0.093			1.951	0.097		
*3 Mo.*	1.175	0.094			2.136	0.098		
*6 Mo.*	1.197	0.098			2.068	0.103		
*12 Mo.*	1.052	0.102			2.118	0.107		

### Outcome and Mediation Analyses

Since a major focus of the paper is mediation of the effects of 12-step attendance on substance use by social variables, we begin with analyses of the effects of 12-step attendance on substance use. In these analyses, 12-step attendance was a significant predictor of both outcomes in the expected direction (p = .015 for PDA and p = .048 for PDHD), suggesting there are effects to mediate; 12-step attendance, however, was not found to significantly predict number of high/low-risk friends in this sample (table 2), and the MacKinnon tests for mediation (Table 3) confirm that neither the number of high-risk friends nor the number of low-risk friends significantly mediated the effect of 12-step on substance use. Nonetheless, the number of high-risk friends and low-risk friends were found to be strong predictors of substance use outcome, in the expected direction, with p-values of.001 or less (table 2).

**Table 2:** Lagged mediation model* of the effects of 12-step meeting attendance (3 m) on social network changes (6 m) and PDA and PDHD (12 m).

*Path*			PDA				PDHD			
			B	SE	t	*p*	B	SE	t	*p*
Direct effect: 12-step attendance predicting PDA/PDHD										
12-Step attendance	→	PDA/PDHD	0.009	0.004	2.44	0.015	−0.002	0.001	−1.99	0.048
Mediational path: 12-step attendance predicting mediators										
12-Step attendance	→	Number of high-risk friends	−0.003	0.003	−1.05	0.297	−0.003	0.003	−1.05	0.297
12-Step attendance	→	Number of low-risk friends	0.006	0.004	1.58	0.116	0.006	0.004	1.58	0.116
Mediational path: mediators predicting PDA/PDHD										
Number of high-risk friends	→	PDA/PDHD	−0.344	0.080	−4.32	0.000	0.059	0.018	3.33	0.001
Number of low-risk friends	→	PDA/PDHD	0.324	0.060	5.39	0.000	−0.064	0.013	−4.89	0.000

*All models controlling for predictors of attrition (education), baseline levels of Percent Days Abstinent (PDA)Percent Days Heavy Drinking (PDHD), baseline levels of the mediator, and predictors of PDA/PDHD (age, gender, commitment to sobriety, motivation, prior hospitalization for alcohol/drug problems and meeting with other mutual help group members outside of meetings at baseline).

**Table 3:** Mediation Testing.

	Sobel Test Statistic	*p*-value
*Percent Days Abstinent*		
High-Risk Friends	0.974	0.330
Low-Risk Friends	1.445	0.148
*Percent Days Heavy Drinking*		
High-Risk Friends	−0.956	0.339
Low-Risk Friends	0.090	0.929

## Discussion

This study examined the social network changes among young adults prior to and following residential SUD treatment and tested whether 12-step MHO participation effects’ on substance use outcomes were mediated via facilitating adaptive changes in the social network. Across the entire network of close friends, there was a significant decrease in high risk members and an increase in low risk members across follow-up. Contrary to predictions and consistent prior findings among adult samples, while both 12-step participation and friend network risk were significantly related to outcomes in expected ways, benefits from participation were not found to be mediated by adaptive changes in 12-step attendees’ social networks. Findings highlight the importance of both close social ties and 12-step MHO participation, underscore the significance of moderating developmental factors, and raise further intriguing questions as to how young adults benefit from 12-step MHOs.

As depicted in figure 2, there was a substantial decline in high risk friends from pre- to post-treatment and a simultaneous, although less substantial, increase in low risk friends. As noted earlier, most SUD treatment programs, including the one in this study, strongly recommend dropping heavy drinkers/drug using individuals in favor of abstainers/infrequent users or, better still, adopting social ties who are already established in recovery themselves. Although it cannot be concluded definitively that treatment is the causal factor at work here, at a minimum, we believe it is likely to have influenced such change. These changes in friend networks are important and were strong predictors of future substance use underscoring the significance of clinical recommendations to reduce involvement with high risk, and increase involvement with low risk, social ties.

Noteworthy and contrary to expectations, social network changes, while themselves strongly predictive of substance use, did not mediate the observed beneficial effects from 12-step participation on outcome. Similar to other studies that have examined life-stage as a moderator of the mechanisms through which AA/NA benefits attendees [33], [35], [36], our findings support consideration of developmental influences in clinical and recovery research [13]. One potential reason for the lack of social mediation found here could be that, given the generally lower prevalence of young adults within AA/NA (only approximately 13–14% of members are under age 30 years old; NA, 2010; AA, 2008), 12-step MHOs, while strongly *encouraging* social network change, may be less able to *facilitate or directly provide access to* new young adult social ties with whom social sober connections can be made, and thereby reducing exposure to substance-related cues, modeling of use, and relapse risk. Young adults in this study appear to be benefitting in other ways from 12-step MHO participation, such as by maintaining or enhancing recovery motivation, self-efficacy, and coping [21], [41]. This should be examined further.

### Limitations

Findings from this study should be considered in light of important limitations. Social influence is an abstract and highly complex construct and while the way we measured and operationalized this in the current study resulted in some significant and interesting findings, we have examined only one aspect of this multifaceted influence. Also, although we used an established measure to capture social network factors (48), we adapted the measure for use with an SUD population and, thus the psychometrics may vary to some degree from the original measure. Furthermore, data were largely self-report and the sample was drawn from a single, private, non-profit, 12-step-oriented residential treatment facility in the mid-Western United States consisting of mostly male, white, young adults. Although the sample and treatment program used in this study has been shown to be fairly representative of both private and public treatment programs and samples [25], generalizations nevertheless should be made cautiously.

### Conclusions

Social influences are important in the onset and offset of substance use and related disorders. Acknowledging this reality, most treatment programs strongly recommend that patients increase the chances of ongoing remission and recovery by reducing involvement with high risk substance using individuals and by increasing involvement with low risk or recovering individuals. One pathway to achieving this goal in adult samples has been via AA/NA participation [13], [51], [52]. Findings here support the value of making recovery-supportive social changes yet, highlight a potentially important developmental difference regarding the ways that young adults may benefit from 12-step participation. While 12-step MHOs may *encourage* social network change, they may be less able to *provide* social network change *directly* for young adults, perhaps because similar-aged peers are less common in MHOs. Findings highlight the importance of both social networks and 12-step MHOs and raise further intriguing questions as to how young adults benefit from 12-step MHOs.
